# Oral Bioavailability and Pharmacokinetics of Sildenafil Orally Disintegrating Tablets under Various Gastric pH Levels Following Administration of Omeprazole in Rats

**DOI:** 10.3390/life13112126

**Published:** 2023-10-27

**Authors:** Chin-Yu Shih, Chao-Yi Chen, Hsien-Te Lin, Ying-Ju Liao, Yao-Jen Liang

**Affiliations:** 1 Graduate Institute of Applied Science and Engineering, Fu-Jen Catholic University, New Taipei City 242062, Taiwan; smufish15@gmail.com; 2 Merdury Biopharmaceutical Corporation, New Taipei City 235030, Taiwan; roger@merdury.com (C.-Y.C.); jason@merdury.com (H.-T.L.); andrea@merdury.com (Y.-J.L.); 3 Institute of Traditional Medicine, School of Medicine, National Yang Ming Chiao Tung University, Taipei 112304, Taiwan

**Keywords:** bioavailability, pharmacokinetics, orally disintegrating tablets, 3D printing technique

## Abstract

Sildenafil citrate, an oral drug used to treat erectile dysfunction, has low water solubility and oral bioavailability. The solubility is greatly influenced by the pH, changing from 37.25 mg/mL to 0.22 mg/mL with a change in pH from 1.2 to 8.0. This indicates that the absorption may decrease in patients who use drugs, such as proton pump inhibitors (PPIs), for gastroesophageal reflux disease. To improve the absorption of sildenafil citrate at various gastric pH levels, a sildenafil citrate orally disintegrating tablet (ODT), which has a rapid disintegration feature, was produced by a 3D printing technique. Our study investigated the pharmacokinetic parameters of the sildenafil citrate ODT in rats after oral administration and compared the absorption of the sildenafil citrate ODT and sildenafil citrate commercial tablet (RLD), with and without PPI treatment. The LC/MS/MS analysis of the plasma sildenafil concentration revealed that the area under curve from time 0 to infinity (AUC_0–∞_) of sildenafil in the sildenafil citrate ODT group was significantly higher than in the sildenafil citrate RLD group whether it was in combination with the PPI or not (274.8% and 144%, respectively; *p* < 0.05). The relative systemic bioavailability of sildenafil citrate RLD significantly decreased with the PPI, but that of sildenafil citrate ODT was not affected by the PPI. These results indicate that the relative systemic bioavailability of sildenafil citrate ODT was increased when it was prepared using the 3D printing technique and the absorption of this formulation was not affected by the PPI.

## 1. Introduction

**Figure 1:**
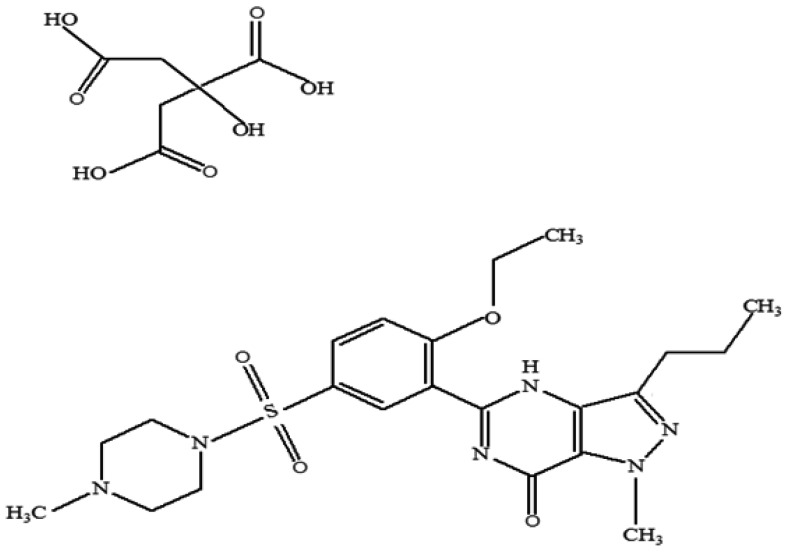
Structure of sildenafil citrate.

Sildenafil citrate is an oral drug used as a therapy for erectile dysfunction [1]. It is designated chemically as 1-[[3-(6,7-dihydro-1-methyl-7-oxo-3-propyl-1 Hpyrazolo [4,3-d] pyrimidin-5-yl)-4-ethoxyphenyl] sulfonyl]-4-methylpiperazine citrate. The sildenafil citrate structure is shown in Figure 1.

Sildenafil citrate is a potent orally active cGMP-specific phosphodiesterase type 5 (PDE5) inhibitor that is effective as a peripheral conditioner in the treatment of male erectile dysfunction (ED) of organic, psychogenic, or mixed etiology [2,3].

Some studies have intended to improve the bioavailability and convenience of sildenafil citrate by changing the dosage form or salt content [4,5]. Sawatdee et al. found that dry foam tablets increased the exposure of sildenafil citrate.

### 1.1. Solubility of Sildenafil Is Affected by the pH

Sildenafil citrate has a basic pKa of 6.5 and an acidic pKa of 9.2. According to research reports [6], sildenafil citrate is a weakly basic drug with a maximum solubility of approximately 37.25 mg/mL at a pH of 1.2 and 37 °C. However, the solubility deceases with an increase in pH. The solubility of sildenafil citrate is approximately 18.53 mg/mL at a pH of 5.0 and 0.22 mg/mL at a pH of 8.0. The solubility profile and data of sildenafil citrate are shown in Table 1.

### 1.2. Sildenafil and Gastroesophageal Reflux Disease (GERD)

Sildenafil citrate is a potent specific PDE5 inhibitor that is used in the treatment of erectile dysfunction. Moreover, during sildenafil clinical trials and post-clinical studies, it has been reported that sildenafil citrate induces dyspeptic symptoms as the most frequent adverse event besides headache and flushing. Therefore, it is conceivable that sildenafil citrate might affect gastric motility [7,8].

Another risk is that sildenafil citrate might predispose a patient to gastroesophageal reflux disorder since it decreases the lower esophageal sphincter (LES) pressure and the strength of peristalsis. In many clinical trials, dyspepsia, a symptom often ascribed to reflux, is one of the major side effects of sildenafil citrate. Although sildenafil citrate does not directly cause GERD, some clinical trials found that, in some patients, there was a decrease in LES pressure [9]. Therefore, in the case of long-term sildenafil citrate use, the risk of GERD may increase under certain dietary conditions.

### 1.3. Proton Pump Inhibitors (PPIs) and Gastroesophageal Reflux Disease (GERD)

Proton pump inhibitors (PPIs), such as omeprazole, lansoprazole, pantoprazole, rabeprazole, and esomeprazole, are widely utilized for the treatment of GERD. PPIs suppress gastric acid secretion by blocking the gastric acid pump H^+^/K^+^-adenosine triphosphatase (ATPase) [10,11].

Omeprazole is one of the most commonly used PPIs, and by effectively reducing gastric acid secretion, it can relieve symptoms such as heartburn, difficulty swallowing, and persistent cough and help prevent esophageal cancer [12]. 

Many patients, including patients with GI problems caused by long-term use of sildenafil citrate, have chosen PPIs to treat gastric ulcers and gastroesophageal reflux.

However, long-term use of PPIs also causes changes in gastric acid secretion and changes in the pH of the gastric acid. As previously discussed, sildenafil citrate solubility is affected by pH. Therefore, there is a problem of whether the absorption of sildenafil citrate will be affected by its combined use with PPIs. Assuming that absorption is greatly affected, the efficacy and safety of sildenafil citrate will be an issue. However, if new formulations can increase the bioavailability of sildenafil citrate and reduce the effect of gastric pH on absorption, these formulations would be a good choice for people who use PPIs.

### 1.4. Three-Dimensional Printing of the Sildenafil Orally Disintegrating Tablet (ODT) Formulation

Since the first 3D-printed drug, SPRITAM^®^ (levetiracetam), was approved by the FDA, 3D printing technology has been widely used in pharmaceuticals.

SPRITAM^®^ tablets have a loose interior and tight exterior, ensuring good mechanical properties and rapid dispersion characteristics. In addition, 3D printing technology can be used to fabricate personalized pediatric preparations for improved compliance [13]. 

**Figure 2:**
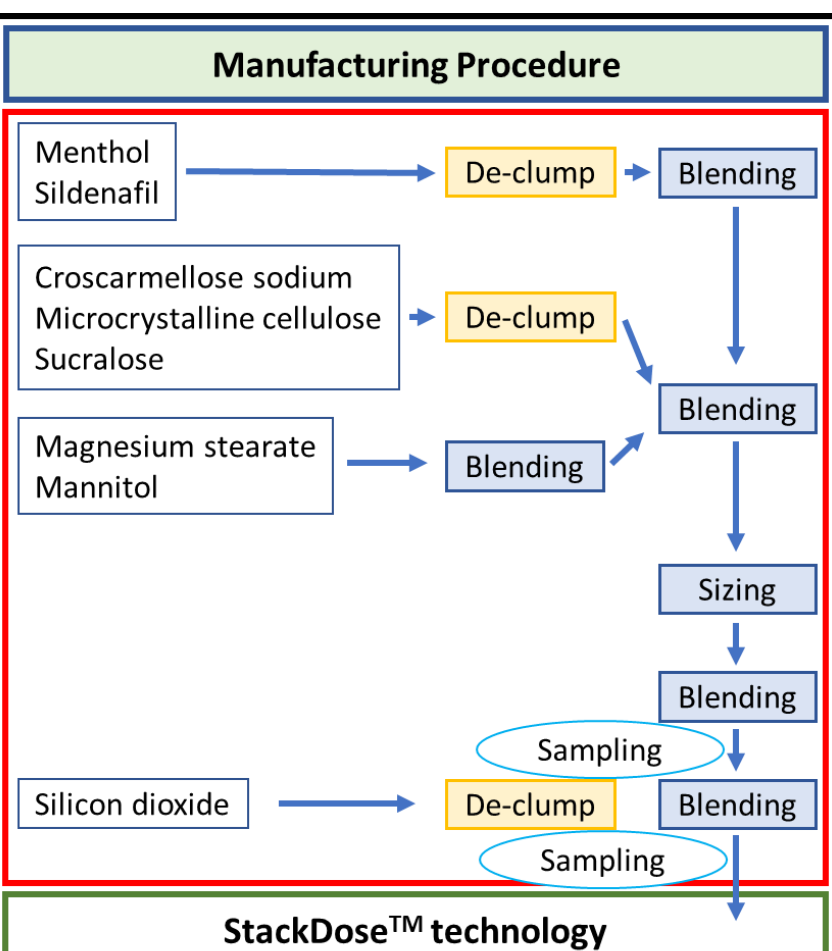
The manufacturing process of sildenafil citrate ODT (orally disintegrating tablet).

Additionally, SPRITAM^®^ is rapidly absorbed (with peak plasma concentrations occurring in about an hour). Merdury Biopharmaceutical utilizes the developed StackDose^TM^ technology platform and uses formulation technology combined with 3D powder bonding equipment to produce rapid-release drugs. Using a 3D printing process makes it easier to achieve the layered release of the tablets, thereby controlling the drug effect time. The manufacturing procedure and consistency are shown in Figure 2. 

A sildenafil citrate 50 mg commercial tablet (Viagra^®^, Pfizer Inc., Fareva Amboise, France) was the first drug approved by the FDA to improve erectile function and is one of the most popular anti-impotence drugs. Conventional sildenafil citrate tablets are typically ingested with a liquid such as water. To provide dosing alternatives for individuals who have difficulty swallowing or have restricted water intake, different oral formulations of sildenafil citrate have been developed, including an orally disintegrating tablet (ODT), sildenafil citrate ODT. In a previous clinical study, bioequivalence was demonstrated for sildenafil citrate ODT administered with or without water relative to sildenafil citrate tablets administered with water and indicated that the absorption of the ODT was not affected by water consumption [14]. The purpose of this study was to further investigate the absorption of the new 3D-printed ODT with or without PPIs. Since the 3D-printed ODTs rapidly disintegrate, they may be more efficiently absorbed and be less affected by the gastric pH.

## 2. Result

### 2.1. Dissolution Test

**Figure 3:**
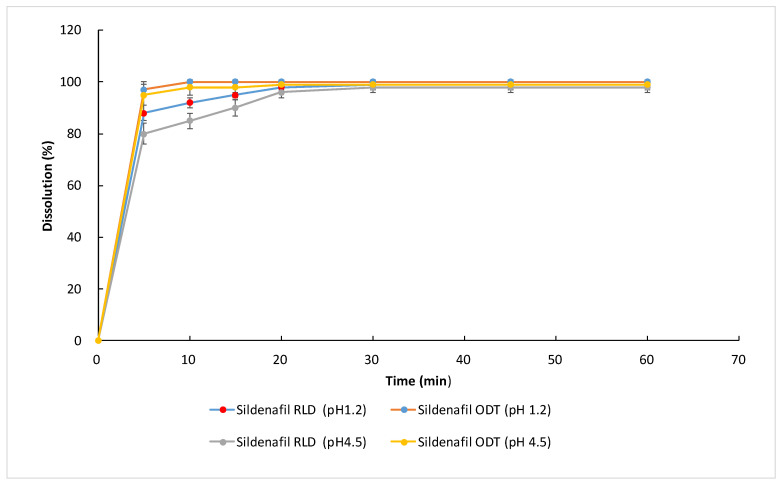
The dissolution rate of sildenafil citrate RLD and ODT at pHs 1.2 and 4.5.

The dissolution test of the sildenafil citrate ODT showed that it dissolved more quickly compared to the RLD, whether at a pH 1.2 or 4.5. (Figure 3). In addition, the dissolution rate of the sildenafil citrate ODT was almost 100% at five minutes. These findings suggested that the sildenafil citrate ODT may have advantages over the sildenafil citrate RLD in absorption at different gastric pHs (1.2 or 4.5).

### 2.2. Gastric pH Changed Following Oral Administration of Omeprazole 

**Figure 4:**
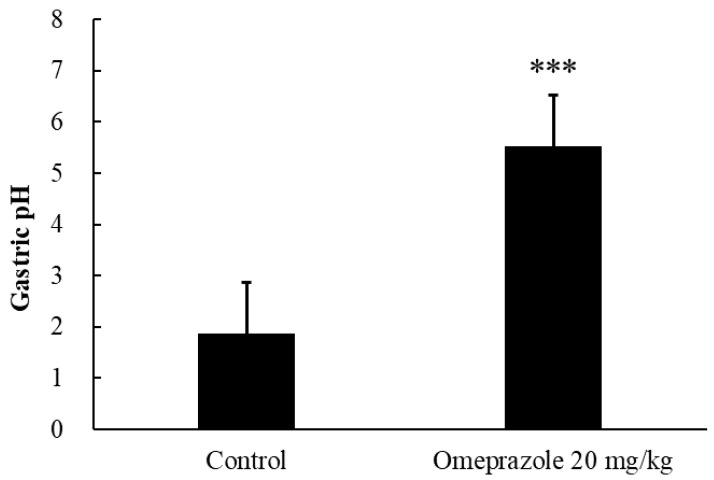
Gastric pH after intraperitoneal injection of omeprazole in rats. Each column represents the mean ± SD (*n* = 6). *** *p* < 0.001.

Animals were sacrificed 2 h after administration of omeprazole or normal saline, the stomach was surgically removed from the euthanized animals, and gastric juices were extracted and immediately centrifuged. The pH of the centrifuged gastric juice supernatant was measured using a pH meter (edge^®^ Dedicated pH/ORP Meter, Blossom, Ltd., Taipei, Taiwan). The measured gastric pH values in each group are presented in Figure 4. 

The gastric pH was 1.88 ± 0.49 (mean ± SD) in the control rats and 5.52 ± 0.55 (mean ± SD) in omeprazole (20 mg/kg) treatment rats, which indicated that intraperitoneal injection of omeprazole significantly increased the gastric pH after 2 h (*p* < 0.001). 

### 2.3. Plasma Concentration and Pharmacokinetic Parameters of ODT and RLD (with and without Pretreatment with Omeprazole)

The dissolution rate of the sildenafil citrate ODT was much faster than that of the sildenafil citrate RLD. The reason may be that the 3D printing sildenafil citrate ODT manufacturing process increased the porosity of the tablet. This finding prompted us to further probe into the bioavailability of the sildenafil citrate ODT in vivo. The study was designed with four arms to investigate the sildenafil citrate ODT or RLD pharmacokinetics following oral administration, with and without pretreatment with omeprazole. The sildenafil concentrations of RLD and ODT in plasma were determined until 24 h. The pharmacokinetic parameters are listed in Table 2. 

The C_max_ and AUC_0–∞_ of the RLD decreased from 92.102 ng/mL and 159.5 ng/mL × h to 44.035 ng/mL and 94.8 ng/mL × h with pretreatment with omeprazole. However, the C_max_ and AUC_0–∞_ of the ODT was not significantly changed whether animals were pretreated with omeprazole or not. This indicated that the absorption of the RLD was significantly influenced by gastric pH, but the absorption of the ODT was not.

We also found that the AUC_0–∞_ of the ODT was higher than that of the RLD (230.9 ng/mL × h vs. 159.5 ng/mL × h or 260.5 ng/mL × h vs. 94.8 ng/mL × h) without and with pretreatment with omeprazole, respectively. These results indicate that the ODT has better bioavailability than the RLD with or without pretreatment with omeprazole, which was in accordance with the dissolution profile. In addition, there was no significant difference in T_1/2_ of the ODT and RLD with or without pretreatment with omeprazole.

### 2.4. The Absorption Effect of Sildenafil with and without Pretreatment with Omeprazole 

A summary of the geometric ratio and bioavailability (F%) of the sildenafil citrate RLD and ODT, with and without pretreatment with omeprazole, is shown in Table 3. Firstly, the bioavailability of the RLD significantly decreased with pretreatment with omeprazole (decreased to 59.4%). This result indicated that a change in gastric pH would influence the absorption of the RLD, which was in accordance with the solubility of sildenafil citrate being affected by the pH value. Secondly, the absorption of the ODT was not significantly influenced by the pretreatment with omeprazole. In addition, the bioavailability of the ODT was significantly higher than the RLD, with and without pretreatment with omeprazole (274.8% and 144%, respectively).

**Figure 5:**
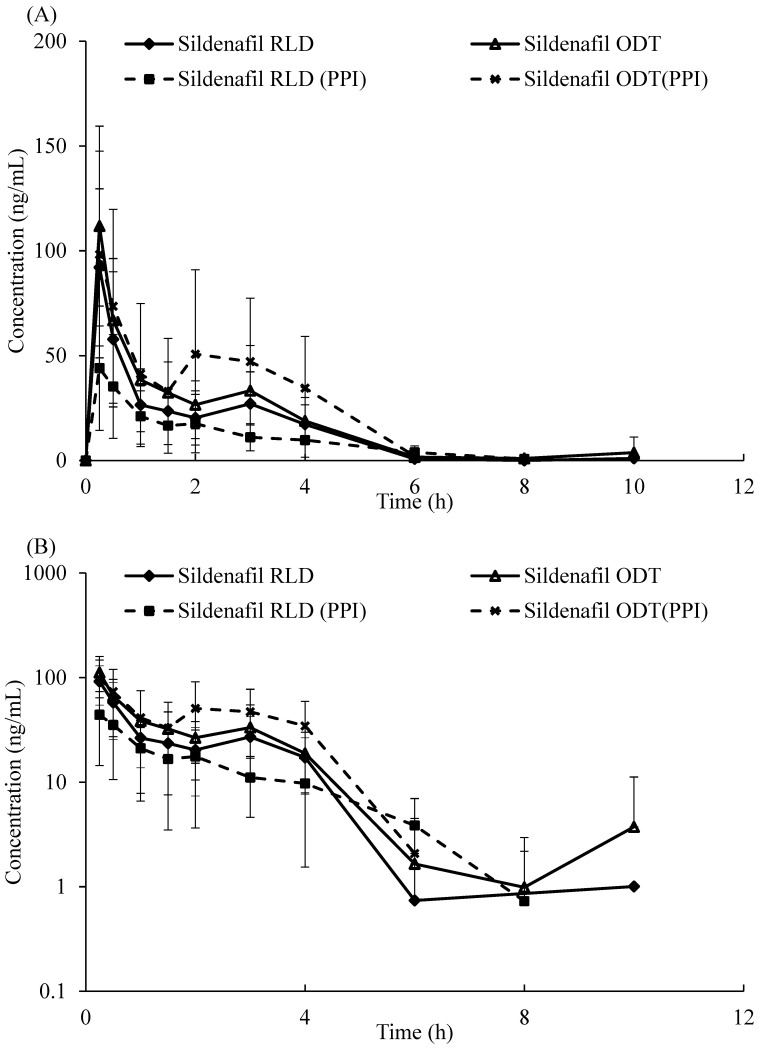
Mean (± SD) plasma concentration–time profiles of sildenafil in rats (*n* = 6) following single dose (20 mg/kg) oral administration of sildenafil citrate ODT or RLD, with and without pretreatment with omeprazole. (**A**) Linear ordinate. (**B**) Log-linear ordinate.

**Figure 6:**
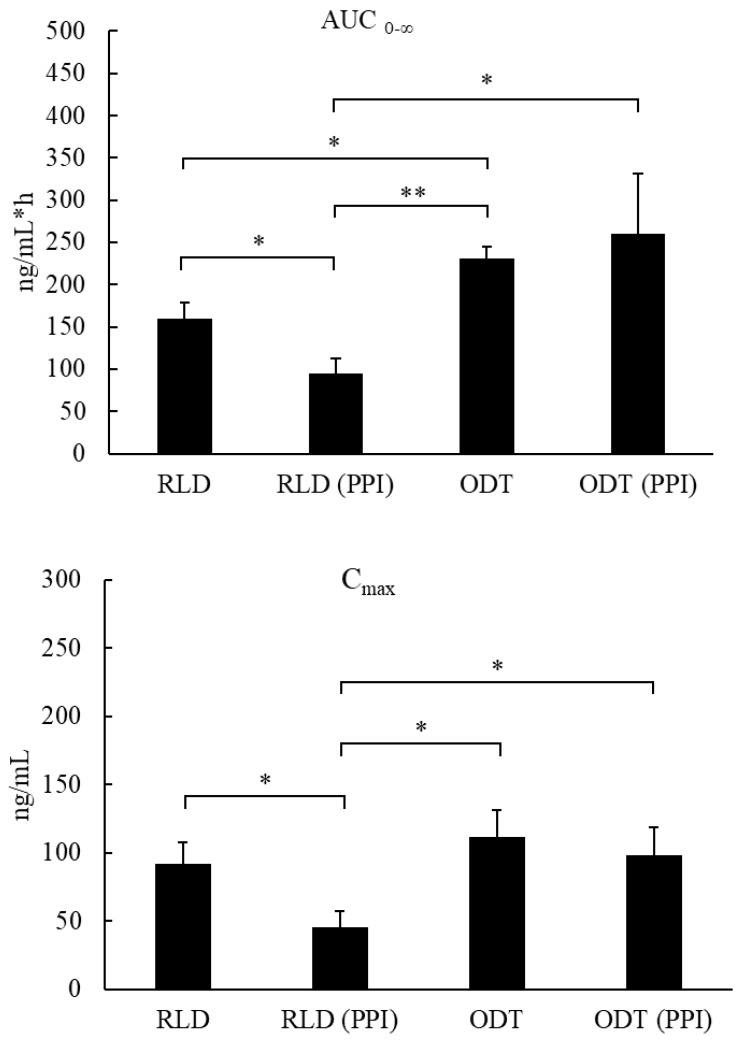
Statistical analysis of pharmacokinetic parameters (AUC_0–∞_ and C_max_) of sildenafil citrate ODT or RLD, with and without pretreatment with omeprazole. Each column represents the mean ± SE (*n* = 6). * *p* < 0.05, ** *p* < 0.001.

Mean plasma concentration–time curves and log-time curves of sildenafil were shown as Figure 5. Pharmacokinetic parameters statistically analysis of sildenafil citrate RLD and ODT, with and without pretreatment with omeprazole, were shown as Figure 6. AUC_0–∞_ of sildenafil in the sildenafil citrate ODT group was significantly higher than in the sildenafil citrate RLD group, with and without pretreatment with omeprazole. (*p* < 0.05).

## 3. Materials

### 3.1. PPIs (Proton Pump Inhibitors)

Omeprazole is one of the most commonly used PPIs and we used omeprazole (Omezol^®^, purchased from Standard Chem & Pharm Co., Ltd., Tainan, Taiwan) to change the gastric pH of rats to mimic that of humans who use PPIs. 

### 3.2. RLD (Sildenafil Citrate Commercial Tablet as Reference Drug)

Sildenafil citrate 50 mg commercial tablets (Viagra^®^, Pfizer Inc., Fareva Amboise, France) were used as the RLD which were obtained using a prescription from a hospital in Taiwan.

### 3.3. ODT (Orally Disintegrating Tablet)

The sildenafil citrate 50 mg ODT (diameter: 13.2 mm; thickness: 6.2 mm) was supplied by Merdury Biopharmaceutical. To improve the absorption of sildenafil citrate, the ODT formulation was made by a 3D printing technique to give it a rapid disintegrating feature. The manufacturing procedure for De-clump involved using a 30 or 40 mesh screen. Blending was performed using a double cone mixer. Sampling was conducted according to international standards, using sampling sticks for the upper, middle, and lower layers. Sizing was determined based on the size of the device.

The list of sildenafil citrate ODT formulation ingredients is shown in Table 4.

### 3.4. Dissolution Test

Before conduction the animal study, the dissolution of the sildenafil citrate ODT and RLD were tested at different pH values (pH 1.2s and 4.5). 

The study was performed using an Agilent Cary 60 UV-Vis Spectrophotometer. Briefly, a tablet sample of the sildenafil citrate RLD or ODT (equivalent to 50 mg of sildenafil citrate) was dispersed in a dissolution basket containing 900 mL of 0.01 N HCl (pH 1.2) or acetate buffer (pH 4.5). The dissolution apparatus was set to run at 100 rpm at 37 ± 0.5 °C. At a predetermined time interval, 5 mL of sample was withdrawn, compensated with fresh media, and analyzed for drug content using UV spectroscopy. Each sample was analyzed in triplicate.

The following equation was used to calculate the percentage of the labeled amount of sildenafil that dissolved:

Dissolution (%) = (A_u_/A_s_) × Cs × V × 1/LC × 100%

where:

Au = absorbance of the sample solution;

As = absorbance of the working standard solution;

Cs = concentration of working standard solution;

LC = label claim (mg/tablet);

V = volume of medium (900 mL).

### 3.5. Animals

Male Sprague Dawley rats (370–410 g) were purchased from BioLASCO Taiwan Co., Ltd., Taipei, Taiwan. The rats were housed in clean polypropylene or corrugated paper cages at a controlled temperature (22 ± 2 °C) and humidity at 40–70% with a 12 h light and dark cycle throughout the experiment. All rats had free access to pelleted food and tap water. Before the experiments, the study protocol were approved by the Institutional Animal Care and Use Committee (IACUC), Rosetta pharmamate co., Ltd., Taiwan.

### 3.6. Influence of Omeprazole on Gastric Acid

The method used was based on a previous report published by Sima et al. [15]. The rats were randomly assigned into two treatment groups. Each group contained six animals. All animals were fasted overnight (about 16 h), and the vehicle control group was administrated normal saline (0.9% sodium chloride) by intraperitoneal injection and the test group was administrated omeprazole 20 mg/kg by intraperitoneal injection. The animals were sacrificed 2 h after the administration of omeprazole, the stomach was surgically removed from the euthanized animals, and gastric juices was extracted and immediately centrifuged. The pH of the centrifuged gastric juice supernatant was measured using a pH meter (edge^®^ Dedicated pH/ORP Meter, Blossom, Ltd., Taipei, Taiwan). The pH meter was previously calibrated at two points using standard solutions of pH 4.0 and pH 6.8.

### 3.7. Sildenafil Citrate ODT or RLD Pharmacokinetics Study with and without Omeprazole

The study was designed with four arms to investigate the sildenafil citrate ODT or RLD pharmacokinetics of oral administration, with and without omeprazole. The rats were divided into four groups with six rats in each group. After overnight fasting (about 16 h), the animals, which were pretreated with and without omeprazole, were orally administrated the sildenafil citrate ODT or RLD.

The tablets (RLD or ODT) were roughly ground into small particles and dispersed in 0.5% methyl cellulose (MC) and mixed homogeneously prior to oral administration. The sildenafil citrate particles were orally administered to rats as a single dose (equivalent to 20 mg/kg sildenafil) using a gastric gavage tube. Omeprazole was administrated as a single dose (20 mg/kg) by intraperitoneal injection 2 h before the oral administration of sildenafil.

Blood samples (0.3 mL) were collected via the tail vein at 0, 0.25, 0.5, 1, 1.5, 2, 3, 4, 6, 8, 10, and 24 h after the oral administration of sildenafil citrate. The blood samples were immediately transferred to heparinized microcentrifuge tubes and centrifuged at 4000 × rpm for 10 min at 4 °C. The separated plasma samples were transferred to Eppendorf tubes and stored at −80 °C until further use.

### 3.8. Preparation of Samples

The plasma samples for the test (25 μL) were spiked with telmisartan (internal standard) at a concentration of 5 ng/100 μL of acetonitrile. Each sample was mixed for 1 min by vortexing and then centrifuged at 13,000 rpm for 5 min. A 100 μL volume of supernatant was diluted with 200 μL of a solution containing acetonitrile, formic acid, and water in a ratio of 5:0.1:95. The sample solution mixture was used for further analysis.

### 3.9. Instrumentation and Chromatographic Conditions

The plasma concentrations of sildenafil were determined using a liquid chromatography–mass spectrometry–mass spectrometry (LC/MS/MS) method; the system suitability was analyzed before the assay. A Waters Alliance 2795 HPLC system (Waters, MA, USA) was used. The HPLC system consisted of a solvent delivery pump equipped with an in-line degasser and an autosampler. The mobile phase used was 30.6% acetonitrile plus 69.4% water containing 0.3% formic acid; the flow rate was 1.0 mL/min (isocratic); and the injection volume was 25 μL. The MS instrument was equipped with an ESI probe and a quadrupole mass analyzer (Micromass Quattro Ultima mass spectrometer system, Manchester, UK). The source temperature was 80 °C, desolvation temperature was 400 °C, the cone voltage was 75 V, and collision energy was 30 V. Nitrogen was used as the nebulizer gas, curtain gas, and collision-activated dissociation gas. Quantification was performed by multiple reaction monitoring of the protonated precursor ion and the related product ion for sildenafil, using the internal standard method with peak area ratios. Sildenafil and telmisartan were eluted at room temperature within 5.5 min. The mass transitions as the [MH]+ molecular ions used for sildenafil and the internal standard were m/z 475.08 → 99.93 and 515.11 → 276.09, respectively, with a dwell time of 0.3 s per transition. The control of the LC/MS/MS system and the data acquisition were performed using the MassLynx V4.0 software (Micromass, Manchester, UK). A calibration curve was prepared in the range of 2.475 ~ 2272.727 ng/mL. Inter-assay precision (CVs%) and accuracy (Relative Error %) of the calibration standards after back-calculation were 1.9% to 11.7% and −5.2% to 3.9%, respectively.

### 3.10. Pharmacokinetics and Statistical Analysis of Data

The plasma concentration versus time curves obtained after each treatment in individual animals were fitted using Microsoft^®^ Excel (version 2013) and are reported as mean ± SD.

The following pharmacokinetic parameters for each animal were analyzed via a non-compartmental model analysis using Phoenix^®^/WinNonlin^®^ (version 9.3): maximum concentration (C_max_), time to reach maximum concentration (T_max_), half-life (T_1/2_), the area under the curve from time 0 to time (t) (AUC_0–t_), and the area under the curve from time 0 to infinity (AUC_0–∞_). The trapezoidal rule was used to calculate AUC_0-∞_. Systemic bioavailability (F) was calculated using the following equation:

F (%)= AUC_0–∞_(test drug)/AUC_0–∞_ (reference)

where AUC of the reference was the AUC of the sildenafil citrate RLD, and AUC (test formulation) was the AUC of the sildenafil citrate ODT. The pharmacokinetic parameters are reported as the mean ± SD. The pharmacokinetic parameters were statistically compared using one-way ANOVA. Mean values were considered significantly different at *p* < 0.05.

## 4. Discussion

Sildenafil citrate is a weakly basic drug and its solubility decreases with increases in pH. In this study, we used omeprazole to change the gastric pH in rats to test the absorption of sildenafil citrate. Pretreatment with omeprazole 2 h before the pH measurements increased the gastric pH to a range that is comparable to the pH seen in humans in the fed state [16]. In another study, Yasumuro et al. also used oral administration of omeprazole to test the effect of PPIs on drug absorption [17]. The gastric pH ranged from 1.53 to 6.8 with omeprazole (100 mg/kg). Although the administration route and dose of omeprazole were different from those of our study, the gastric pH had a similar change from low to high.

In our study, the bioavailability of the RLD significantly decreased with the pretreatment with omeprazole (decreased to 59.4%). This result indicated that people who use omeprazole may have a relatively lower bioavailability of the RLD and a reduced anti-impotence effect. In contrast, the sildenafil citrate ODT had better absorption than the sildenafil citrate RLD in rats pretreated with omeprazole. When combined with omeprazole, the C_max_ and AUC_0–∞_ of the ODT were higher than those of the RLD (geometric ratios were 221.2% and 237.0%, respectively). This result indicated that the sildenafil citrate ODT performed better than the sildenafil citrate RLD under higher gastric pH conditions. 

A previous study investigated the dissolution profile of three ODT formulations (with different taste-masking film layers consisting of amino alkyl methacrylate copolymer, polyvinylacetal diethylaminoacetate, and ethylcellulose) [18]. Their results indicated that different methods and coating polymers also have strong impacts on the drug dissolution. 

In our study, the sildenafil citrate ODT was coated on the outside with menthol to change the flavor of the sildenafil citrate ODT. In the inner layer, mannitol was used. Both menthol and mannitol were reported to enhance the solubility and dissolution of the Biopharmaceutics Classification System (BCS) Class II drugs sulfamethoxazole [19] and clotrimazole [20], respectively. Sildenafil citrate is a BCS Class II drug [21], which helps to explain the better absorption rate of our sildenafil citrate ODT compared to the RLD.

In our dissolution test at different pH levels (1.2 and 4.5), we found that the dissolution rate of the sildenafil citrate RLD at pH 4.5 was slower than that at pH 1.2. However, the dissolution rate of the sildenafil citrate ODT did not significantly change (from pHs 1.2 to 4.5). The dissolution rate could also help to explain the better absorption rate of the sildenafil citrate ODT. Our results are in accordance with the study by Kim [22]. Using the in vitro dissolution profile as input data, the final IVIVC (in vivo–in vitro correlation) model could successfully predict the complex in vivo pharmacokinetic profiles of sildenafil.

Considerations for the clinical use of PPIs with sildenafil citrate are important since many GERD patients also take Viagra^®^ (sildenafil citrate RLD) to improve erectile function. Long-term use of PPIs (e.g., omeprazole) can change the gastric pH from 2.0 to over 6.0, a 10,000-fold change [23], and the absorption of sildenafil citrate may decrease compared with normal people. Since the efficacy of anti-impotence is relative to the exposure of sildenafil in the circulating blood, the efficacy decreases following a decreased exposure. This is an issue for people on long-term PPI therapy and using the sildenafil RLD for anti-impotence. If the efficacy of the sildenafil RLD becomes lower, they will need to take more drugs for the anti-impotence effect. This increase in anti-impotence drugs could result in some safety and cost-related issues. 

Our study found that the absorption of the sildenafil citrate ODT was not significantly affected by the pretreatment with omeprazole. Unlike the sildenafil citrate RLD, the T_max_ and bioavailability of the sildenafil citrate ODT were not changed with the pretreatment with omeprazole. People using PPIs long-term would not need to adjust the dose of sildenafil citrate to maintain the efficacy. The sildenafil citrate ODT might be a good choice for people who use PPIs long-term and need sildenafil citrate to treat erectile dysfunction. 

## 5. Conclusions

The relative systemic bioavailability of the sildenafil citrate ODT was increased by using the 3D printing technique and the absorption of this formulation was not affected by PPIs. In addition to sildenafil citrate, the solubility or absorption of certain drugs is affected by gastric pH. The 3D printing technique could be applied to their formulation development.

## Figures and Tables

**Table 1:** Solubility (mg/mL) of sildenafil citrate at different pHs.

pH	Solubility (mg/mL) in 37 °C Water
1.2	37.25
4	21.19
5	18.53
6.8	0.4
7.4	0.24
8	0.22

**Table 2:** Pharmacokinetic parameters of sildenafil following single dose (20 mg/kg) oral administration of sildenafil citrate RLD or ODT, with and without pretreatment with omeprazole. (*n* = 6).

RLD					
Parameters	AUC_0–t_	AUC_0–∞_	C_max_	T_max_	T_1/2_
	(ng/mL × h)	(ng/mL × h)	(ng/mL)	(h)	(h)
Mean	126.1	159.5	92.102	0.94	1.90
SD	33.2	38.8	37.486	1.38	1.35
CV(%)	26.3	24.3	40.7	146.8	71.1
Geometric Mean	122.3	155.3	85.353	0.57	1.47
Maximum	162.2	198.3	133.953	3.00	3.66
Minimum	86.4	107.6	49.867	0.25	0.55
Median	127.9	169.4	92.295	0.38	1.64
**ODT**					
**Parameters**	**AUC_0–t_**	**AUC_0–_**_∞_	**C_max_**	**T_max_**	**T_1/2_**
	**(ng/mL × h)**	**(ng/mL × h)**	**(ng/mL)**	**(h)**	**(h)**
Mean	167.2	230.9	111.823	0.25	2.79
SD	73.2	33.3	47.649	0.00	1.13
CV(%)	43.8	14.4	42.6	0.0	40.4
Geometric Mean	153.5	228.9	103.873	0.25	2.58
Maximum	262.2	267.5	180.070	0.25	4.04
Minimum	88.7	193.5	64.174	0.25	1.39
Median	158.9	231.3	95.709	0.25	2.86
**RLD(PPI)**					
**Parameters**	**AUC_0–t_**	**AUC_0–_**_∞_	**C_max_**	**T_max_**	**T_1/2_**
	**(ng/mL × h)**	**(ng/mL × h)**	**(ng/mL)**	**(h)**	**(h)**
Mean	72.8	94.8	44.035	1.13	2.36
SD	36.8	42.2	29.625	1.15	1.60
CV(%)	50.6	44.5	67.3	102.1	67.6
Geometric Mean	63.2	85.0	35.137	0.67	1.89
Maximum	109.6	137.0	80.202	3.00	4.53
Minimum	25.1	35.4	13.932	0.25	0.76
Median	81.3	105.0	40.785	0.63	2.04
**ODT(PPI)**					
**Parameters**	**AUC_0–t_**	**AUC_0–_**_∞_	**C_max_**	**T_max_**	**T_1/2_**
	**(ng/mL × h)**	**(ng/mL × h)**	**(ng/mL)**	**(h)**	**(h)**
Mean	230.2	260.5	98.251	0.44	2.07
SD	185.0	175.0	49.279	0.38	1.66
CV(%)	80.4	67.2	50.2	86.4	80.3
Geometric Mean	155.9	201.3	84.781	0.40	1.44
Maximum	483.4	486.3	174.165	1.00	4.19
Minimum	40.4	60.2	42.382	0.25	0.52
Median	198.4	247.7	87.570	0.25	1.79

**Table 3:** The geometric ratio and bioavailability (F%) of sildenafil following single dose (20 mg/kg) oral administration of sildenafil citrate ODT or ODT, with and without pretreatment with omeprazole (*n* = 6).

Sildenafil Citrate				
	ODT *	RLD * (with PPI)	ODT * (with PPI)	ODT ** (with PPI)
C_max_ Geometric ratio (90 % CI)	121.7 (77.8~190.3)	41.2 ( 20.9~81.6)	91.1 (50.7~163.5)	221.2 (102.4~477.7)
AUC_0–t_ Geometric ratio (90 % CI)	125.5 (84.4~186.6)	51.7 (31.3~85.5)	127.4 (56.3~288.5)	246.4 (98.7~615.5)
AUC_0–∞_ Geometric ratio (90 % CI)	147.4 (118.3~183.7)	54.7 (34.8~85.9)	129.7 (66.9~251.2)	237.0 (111.6~503.3)
Bioavailability F (%) ***	144.0	59.4	163.0	274.8

* Compared to RLD (without PPI). ** Compared to RLD (with PPI). *** F (%) = mean AUC_0–∞_(test drug)/mean AUC_0–∞_ (reference).

**Table 4:** All ingredients of sildenafil citrate ODT formulation in this study.

Ingredient	Amount (%)	Company	Country
Sildenafil citrate	14.05	Heteor	Telangana, India
Mannitol	69.85	ROQUETTE	Beinheim, France
AVICEL PH-102 NF microcrystalline cellulose	5.6	DuPont Nutrition	Wilmington, United States of America
Ac-Di Sol^®^ SD711 NF croscarmellose sodium	6	DuPont Nutrition	Wilmington, United States of America
Silicon dioxide (Sylysia #350)	0.5	Fuji Silysia	Kasugai-shi, Japan
Magnesium stearate	2	Peter Greven Asia Sdn. Bhd.	Penang, Malaysia
Sucralose	1	Merck	Darmstadt, Germany
Menthol	1	BASF	Ludwigshafen, Germany

## Data Availability

Not applicable.
